# A Climatitic Distemperature

**Published:** 1864-09

**Authors:** 


A Climatitic Distemperature, extending over a large portion of the earth's surface, and concurrent with epidemic manifestations, has been noted during the last twelve months, and indeed for a longer period. The drought of the last winter appears to have been dependent on causes operating almost universally. It was felt northward in the latitude of Vancouver's Island, and in the central region east of the Rocky Mountains, in Brazil, and in different parts of central and southern Europe. On the other hand, the spring months have been marked by heavy and unusual rains in the southern counties of California, in Arizona, and in the region of the Sierra Nevada Mountains. The spring winds have been remarkably turbulent in all directions, on the Pacific coast of North America. Withering siroccos have visited the southern section of California, and snow-storms the mono country to the eastward. There is something wrong. In the present condition of our knowledge we may not detect the relation of atmospheric to epidemic influences. But that they are related one can scarcely doubt. Two winters ago we had deluges and small-pox. The climate extremes, then and now, undoubteldy belong to the same atmospheric cycle. In like manner, we may presume that the variolous epidemic of two years ago, and the subsequent diptheritic, typhoid and petechial tendency belong to one epidemic cycle. The subject presents an immense field for exploration and discovery.—*San Francisco Medical Press.*

